# Apolipoprotein C-I is an *APOE* genotype-dependent suppressor of glial activation

**DOI:** 10.1186/1742-2094-9-192

**Published:** 2012-08-10

**Authors:** Eiron Cudaback, Xianwu Li, Yue Yang, Thomas Yoo, Kathleen S Montine, Suzanne Craft, Thomas J Montine, Christopher Dirk Keene

**Affiliations:** 1 Department of Pathology, University of Washington, Seattle, WA, 98104, USA; 2 Department of Psychiatry and Behavioral Sciences, University of Washington, Seattle, WA, 98195, USA; 3 Geriatric Research, Education, and Clinical Center, Veterans Affairs Puget Sound Health Care System, Seattle, WA, 98108, USA

**Keywords:** ApoE, ApoC-I, Alzheimer’s disease, Cerebrospinal fluid, Targeted replacement mice

## Abstract

**Background:**

Inheritance of the human ϵ4 allele of the apolipoprotein (apo) E gene (*APOE*) significantly increases the risk of developing Alzheimer’s disease (AD), in addition to adversely influencing clinical outcomes of other neurologic diseases. While apoE isoforms differentially interact with amyloid β (Aβ), a pleiotropic neurotoxin key to AD etiology, more recent work has focused on immune regulation in AD pathogenesis and on the mechanisms of innate immunomodulatory effects associated with inheritance of different *APOE* alleles. *APOE* genotype modulates expression of proximal genes including *APOC1*, which encodes a small apolipoprotein that is associated with Aβ plaques. Here we tested the hypothesis that *APOE*-genotype dependent innate immunomodulation may be mediated in part by apoC-I.

**Methods:**

ApoC-I concentration in cerebrospinal fluid from control subjects of differing *APOE* genotypes was quantified by ELISA. Real-time PCR and ELISA were used to analyze apoC-I mRNA and protein expression, respectively, in liver, serum, cerebral cortex, and cultured primary astrocytes derived from mice with targeted replacement of murine APOE for human *APOE* ϵ3 or ϵ4. ApoC-I direct modulation of innate immune activity was investigated in cultured murine primary microglia and astrocytes, as well as human differentiated macrophages, using specific toll-like receptor agonists LPS and PIC as well as Aβ.

**Results:**

ApoC-I levels varied with *APOE* genotype in humans and in *APOE* targeted replacement mice, with ϵ4 carriers showing significantly less apoC-I in both species. ApoC-I potently reduced pro-inflammatory cytokine secretion from primary murine microglia and astrocytes, and human macrophages, stimulated with LPS, PIC, or Aβ.

**Conclusions:**

ApoC-I is immunosuppressive. Our results illuminate a novel potential mechanism for *APOE* genotype risk for AD; one in which patients with an ϵ4 allele have decreased expression of apoC-I resulting in increased innate immune activity.

## Background

Late onset or sporadic Alzheimer’s disease (AD) is a progressive neurodegenerative disorder and the most common cause of dementia [1]. The pathologic hallmark of AD is cerebral deposition of senile plaques composed primarily of aggregated amyloid β (Aβ), a pleiotropic neurotoxin known to activate resident microglia, the principle innate immune effector cells of the brain. Indeed, cellular markers for activated neuroinflammation are readily detectable in the brain of patients with AD and extensively co-localize with amyloid β (Aβ) plaques [2]. While neuroinflammation may be neuroprotective or neurotoxic depending on the type and duration of response, there is mounting genomic and experimental evidence that chronic inflammatory processes contribute to neurodegeneration through impaired clearance of Aβ peptides, enhanced production of cytotoxic cytokines including TNF-α, IL-1β, and IL-6, and generation of reactive oxygen species as reviewed by Galasko and Montine [3]. The identification of molecules that modulate innate immune activation in AD and other neurodegenerative disease is needed to identify potential therapeutic targets; a strong candidate molecule is apolipoprotein E (apoE).

ApoE is abundantly synthesized by the liver, acting predominantly as a constituent of circulating very low density lipoprotein (VLDL), although a distinct apoE pool exists in the central nervous system (CNS) [4] that is synthesized primarily by astrocytes and is found in high-density lipoprotein (HDL)-like particles [5]. In humans, apoE exists as three isoforms, apoE2, apoE3, and apoE4, encoded by the *APOE* alleles ϵ2, ϵ3, and ϵ4, respectively. Inheritance of the *APOE* ϵ4 allele represents the single greatest genetic risk factor for development of late-onset AD [6]. While this strong genetic association relates disease risk and molecular relevance, it fails to provide clear evidence for underlying disease mechanisms. Elegant studies have demonstrated the importance of differential apoE isoform lipidation status on modulation of Aβ peptide metabolism and trafficking in transgenic mouse models, and thereby a mechanism by which inheritance of *APOE* ϵ4 may increase the risk of developing AD [7,8]. ApoE is known to bind to Aβ [9] in an isoform-dependent manner [10]. *APOE* genotype also has been associated with disease risk or clinical outcome of other neurodegenerative diseases such as vascular dementia, Parkinson’s disease, and dementia with Lewy bodies as described in a recent review [11], suggesting mechanisms, in addition to modulation of Aβ trafficking, by which apoE isoforms may influence neurodegenerative processes. Common to this diverse group of neuropathologies is innate immune activation [12-14]. Indeed, *APOE* genotype is associated with regulation of peripheral immunity [15], including progression of HIV infection [16], and differentially regulates innate immune function in microglia and astrocytes through modulation of microglia migration, microglia and astrocyte pro-inflammatory cytokine production in response to toll-like receptor (TLR) activators, and formation of reactive oxygen species [17-19]. Furthermore, transgenic mice expressing the human ϵ4 allele show reduced apoE levels compared to ϵ3 animals [20], suggesting a possible mechanism for the apoE isoform-specific regulation of CNS cytokine secretion observed *in vivo*[21].

*APOE* genotype influences expression of nearby genes, including *APOC1*[22], with *APOC1* polymorphisms linked to *APOE* ϵ4 suggested as possible risk factors for AD [23]. Indeed, *APOC1* is part of the *APOE/C-I/C-IV/C-II* gene cluster on the long arm of chromosome 19 [24]. Apolipoprotein C-I (apoC-I) is a small 6.6 kD apolipoprotein constituent of HDL that is known to inhibit receptor-mediated lipoprotein clearance, especially particles containing apoE, via direct blockade of the low density lipoprotein (LDL) and VLDL receptors and LDL receptor-related protein [25]. Like most apolipoproteins, apoC-I is synthesized predominantly by liver, with CNS pools significantly lower than apoE [5] and likely derived from astrocyte expression [26,27]. Regulation of apoC-I expression is complex and includes linkage disequilibrium of the H2 polymorphism of *APOC1* with the ϵ4 allele of *APOE*. Because of this, *APOC1* allelic variation has been proposed as a significant risk factor for AD [28,29]. Moreover, there is apparent biologic plausibility since apoC-I co-localizes with Aβ plaques in brain in AD [27], and frontal cortex apoC-I levels are reduced in patients with AD [26], suggesting a possible role of apoC-I in the pathogenesis of the disease.

We hypothesized that differential expression of apoC-I depending on *APOE* genotype could represent a novel mechanism for *APOE* genotype-associated risk for neurodegenerative diseases. In the current study we sought to identify *APOE* genotype differences in apoC-I expression, and whether apoC-I had immunomodulatory functions akin to apoE. We propose that the apoE isoform-dependent effects on innate immune modulation are at least in part the result of *APOE* genotype-dependent differences in the levels of apoC-I, a novel suppressor of innate immune activation in the CNS.

## Methods

### Materials and reagents

Double-stranded polyinosinic-polycytidylic acid (PIC), phorbol 12-myristate 13-acetate (PMA), and Aβ_1-42_ were purchased from Sigma-Aldrich (St. Louis, MO, USA); lipopolysaccharide (LPS) was purchased from Calbiochem (La Jolla, CA, USA); Pam_3_CSK_4_ (Pam3) and CpG oligonucleotides were purchased from Invivogen (San Diego, CA, USA); low endotoxin recombinant human RAP (receptor associated protein) was purchased from Innovative Research (Novi, MI, USA).

### Human cerebrospinal fluid (CSF)

CSF was collected from male and female participants who were 65 years of age or older, cognitively normal as determined by a comprehensive neuropsychologic test battery [30], and free of major psychiatric and neurologic disorders, substance abuse, renal, hepatic, pulmonary, and cardiovascular disease, who were enrolled in a recent intervention study [30]. Cerebrospinal fluid (CSF) was obtained by lumbar sac puncture before assignment to treatment group; CSF was collected prior to 10:00 AM following a 12-h fast. CSF was aliquoted, flash frozen on dry ice, and stored at −70°C prior to assay. The Human Subjects Review Committees of the University of Washington and the Veterans Affairs Puget Sound Health Care System approved the study, and written informed consent was obtained from all participants. All CSF samples were de-identified and coded so that all analyses were performed blinded.

### Animals

Wild type (WT) C57BL/6 mice were purchased from the Jackson Laboratory (Bar Harbor, ME, USA). Mice with homozygous targeted replacement (TR) of mouse apolipoprotein E gene with human ϵ2 (TR APOE2), ϵ3 (TR APOE3), or ϵ4 (TR APOE4) were the generous gift of Dr. Maeda [31,32]. In brief, these TR mice have the coding exons of mouse *apoE* replaced with those from human *APOE* and express full-length human apoE2, apoE3, or apoE4 under control of the appropriate regulatory elements. Original strains were backcrossed greater than six generations to a C57BL/6 genetic background. All mice were housed in a temperature-controlled specific pathogen-free facility with a strict 12-h light/dark cycle and with free access to food and water, and used with approval of the University of Washington Animal Care and Use Committee.

### Cell culture

Primary murine mixed glial cultures were generated from 0- to 3-day old pups as previously described [17]. In brief, cerebral cortices were homogenized and plated in 162-cm^2^ flasks (Corning Inc., Corning, NY, USA) coated with poly-L-ornithine (Sigma-Aldrich) and maintained at 37°C and 5% CO_2_ in DMEM/F-12 supplemented with 10% fetal bovine serum (FBS) (HyClone, Logan, UT, USA), 100 U/ml penicillin, and 100 μg/ml streptomycin (Invitrogen, Carlsbad, CA, USA). Microglia were isolated from mixed glial cultures by gentle agitation and sub-cultured in 96-well plates (BD Biosciences, Bedford, MA, USA) at 5 × 10^4^ cells/well in serum-free media prior to treatment. Astrocyte cultures were generated via enzymatic detachment of mixed glial cultures with trypsin (Invitrogen) and subculture to 96-well plates. Prior to treatment, astrocyte cultures (> 95% pure as identified by immunostaining for glial fibrillary acidic protein) were allowed to reach confluence (3 d) and subsequently treated as described for microglial cultures. The human monocytic suspension cell line THP-1 was maintained in RPMI-1640 (Invitrogen) supplemented with 10% FBS, penicillin/streptomycin, and 0.05 mM 2-mercaptoethanol (Sigma-Aldrich). Subculture of THP-1 cells to 6-well plates (BD Biosciences) at 4 × 10^5^ cells/well was followed by 3 d treatment with 50 ng/ml PMA (Sigma-Aldrich) for differentiation to adherent macrophage phenotype. Cells were washed three times with PBS to remove non-adherent cells, and then media replaced with serum-containing media for 24 h to reduce PMA-induced background cytokine synthesis. Serum-free media was used for all cell treatments.

### ELISA and Luminex

Murine IL-6 and TNF-α in medium from treated microglia and astrocytes were quantified by ELISA (R & D Systems, Minneapolis, MN, USA). Levels of 32 mouse cytokines/chemokines in medium were determined using a custom mouse MILLIPLEX MAP kit that measures eotaxin, granulocyte-colony stimulating factor (G-CSF), granulocyte macrophage-colony stimulating factor (GM-CSF), IFNγ, IL-1α, IL-1β, IL-2, IL-3, IL-4, IL-5, IL-6, IL-7, IL-9, IL-10, IL-12(p40), IL-12(p70), IL-13, IL-15, IL-17, interferon gamma-induced protein 10 (IP-10), keratinocyte-derived chemokine (KC), leukemia inhibitory factor (LIF), lipopolysaccharide-induced CXC chemokine (LIX), monocyte chemotactic protein-1 (MCP-1), macrophage-colony stimulating factor (M-CSF), monokine induced by gamma interferon (MIG), macrophage inflammatory protein (MIP)-1α, MIP-1β, MIP-2, regulated upon activation normal T-cell expressed and secreted (RANTES), TNF-α, and vascular endothelial growth factor (VEGF) (Millipore, Billercia, MA, USA). Human CSF and mouse serum from targeted replacement mice were assayed for apoC-I using a previously described [33] human apoC-I sandwich ELISA that recognizes both human and murine proteins, showing no cross-reactivity in *apoc1*^*−/−*^ mice. Briefly, polyclonal goat anti-human apoC-I capture and HRP-conjugated detector antibodies (Academy Biomedical Co., Houston, TX, USA) were used to determine apoC-I concentrations from diluted human (1:80) and mouse (1:20) biological samples.

### qPCR

Age-matched (12 week old) TR *APOE* mice were anesthetized and transcardially perfused with ice-cold PBS. The liver and whole brain were harvested, and cerebral cortices rapidly dissected, flash frozen in liquid nitrogen, and stored at −80°C until processed. Total RNA was isolated from liver, brain, cultured primary astrocytes, and THP-1 cells using an RNeasy extraction kit (Qiagen, Valencia, CA, USA) according to the manufacturer’s protocol. RNA (1 μg) was reversed-transcribed using an Advantage RT-for-PCR kit (Clontech, Mountain View, CA, USA), and quantitative expression of mouse apoC-I mRNA was determined by qPCR using gene-specific TaqMan Gene Expression Assays run on an ABI 7900HT (Applied Biosystems, Carlsbad, CA). Human IL-6 and TNF-α mRNA expression in THP-1 cells (see above) was determined using the same protocol. Quantification of gene expression was calculated using the delta-delta cycle threshold (ΔΔct) method with normalization to 18 S rRNA for murine samples and GAPDH for THP-1 cells.

### Statistical analysis

All statistical analyses were performed using GraphPad Prism 5.0 (GraphPad Software Inc., San Diego, CA, USA).

## Results

### *APOE* ϵ4 is associated with lower human CSF apoC-I concentration and decreased apoC-I expression in TR APOE4 mice

**Figure 1:**
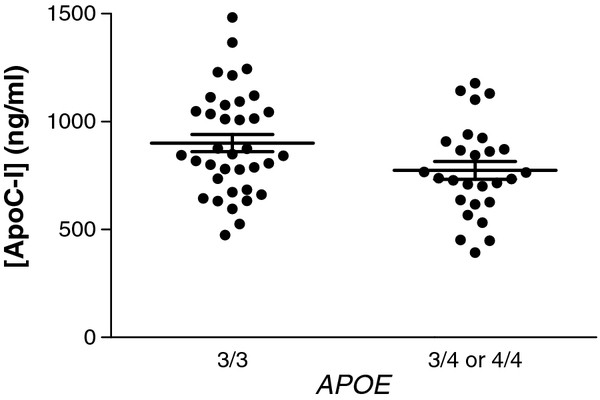
**Cerebrospinal fluid (CSF) apoC-I is reduced in human subjects carrying an*APOE*ϵ4 allele.** CSF obtained via lumbar sac puncture from normal volunteers 65 years or older who enrolled in two recent intervention studies was assayed for apoC-I (n = 63). CSF was collected from initial lumbar tap prior to treatment protocol assignment. Results were stratified by *APOE* with 36 individuals homozygous for *APOE* ϵ3 and 27 individuals either homozygous for *APOE* ϵ4 or heterozygous *APOE* ϵ3 and ϵ4. There were too few (n = 7) individuals in this data set with *APOE* ϵ2. Individuals with an *APOE* ϵ4 allele had approximately 15% lower CSF apoC-I than individuals homozygous for *APOE* ϵ3 (*P* = 0.02).

One group has reported that apoC-I mRNA is consistently lower in frontal cortex of brains from control and AD patients that harbor the *APOC1* H2 allele (usually inherited with APOE ϵ4) compared with patients with the *APOC1* H1 allele (usually inherited with *APOE* ϵ3); however, while human brain regional apoC-I protein in controls was also lower in association with *APOC1* H2 allele the opposite association was observed in patients with AD [26]. These observational data are complex because *APOEAPOC1*, and disease state each varied, and important potential confounders like post mortem interval and agonal state were not reported. Therefore, we first sought a more direct determination of whether human CNS levels of apoC-I varied with *APOE* genotype. CSF from pretreatment assignment lumbar taps in 63 normal volunteers (aged 65 and older) enrolled in a recent clinical investigation [30] contained significantly less (*P* = 0.02, Students *t*-test) apoC-I in those carrying at least a single copy of *APOE* ϵ4 (774 ± 41 ng/mL) compared to those homozygous for *APOE* ϵ3 (901 ± 40 ng/mL) (Figure 1). These data extend to the CNS observations already made in the periphery that while the *Hpa I* restriction site insertional *APOC1* variant (the so-called H2 polymorphism) is observed to be in linkage disequilibrium with *APOE* genotype, a strong correlation exists between carriers of the *APOE* ϵ4 allele and reduced plasma apoC-I expression [34].

**Figure 2:**
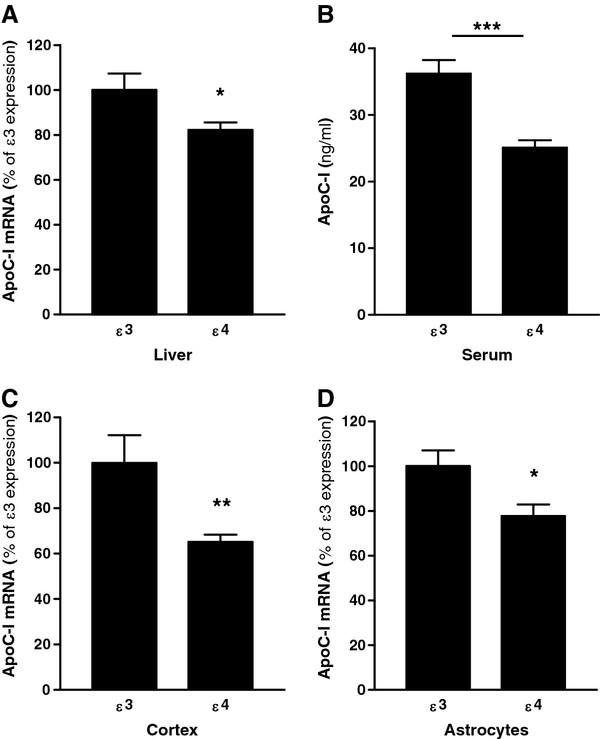
**ApoE genotype influences apoC-I expression in humanized mice.** Liver (**A**), serum (**B**), and cerebral cortex (**C**) were collected from 12 week-old targeted replacement *APOE* mice homozygous for ϵ3 or ϵ4. Total RNA was isolated from liver (**A**) and cortex (**C**) and qPCR performed to quantify apoC-I mRNA expression from each genotype. Serum (**B**) was assayed for apoC-I expression using ELISA. (**D**) Total RNA was isolated from primary astrocyte cultures prepared from humanized *APOE* mice, and qPCR performed to quantify apoC-I mRNA expression from each genotype. Expression of apoC-I from all tissues analyzed was significantly lower in ϵ4 animals compared to ϵ3. Data are expressed as the mean ± standard error of the mean (SEM) percentage apoC-I mRNA (**A**, **C**, **D**) of ϵ3 mice or protein concentration (B); n = 4 to 6. *P < 0.05; **P < 0.01; ***P < 0.001; Student’s *t*-test.

To confirm our observation from human CSF and simultaneously to eliminate the human H2 polymorphism confounder, we investigated apoC-I expression in TR APOE mice. Quantitative PCR analysis of total RNA isolated from liver of TR APOE mice showed significant reduction (approximately 18%) in apoC-I expression in TR APOE4 mice compared to TR APOE3 animals (Figure 2a), and circulating levels of apoC-I also were found to be significantly reduced (> 30%) in serum from the same mice (Figure 2b). ApoC-I expression in cerebral cortex and primary astrocyte cultures from cortex of TR APOE4 mice also was similarly significantly reduced compared to TR APOE3 mice (Figure 2c and d), confirming astrocyte expression of apoC-I [26] and its association with *APOE*. Indeed, these data support previous reports that *APOE* genotype modulates the expression of other constituents of the *APOE/C-I/C-IV/C-II* gene cluster [35].

### ApoC-I modulates innate immune responses to TLR agonists in murine microglia and astrocytes

Because apoC-I levels were lower in human CSF in adult *APOE* ϵ4 carriers, and apoC-I expression was lower in TR APOE4 mouse cerebral cortex and primary astrocytes, we hypothesized that concentration-dependent actions of apoC-I might explain in part some of the innate immune regulation associated with *APOE* genotype. While apoC-I was originally identified as an inhibitor of apoE-containing lipoprotein uptake [36], various apolipoproteins also have been implicated in the modulation of both peripheral and central innate immune system responses [37,38]. We hypothesized that apoC-I might also have an innate immunomodulatory function and tested this by investigating the influence of apoC-I on TLR-dependent cytokine and chemokine secretion from cultured primary microglia or astrocytes. We screened for apoC-I-altered expression of multiple innate immune effectors in pooled medium from two independent experiments (3 replicates each) using WT primary murine microglia cultures stimulated for 18 h with the TLR3 agonist PIC, a potent innate immune activator known to elicit robust microglial cytokine expression and secretion [39]. Of the 32 analytes measured, PIC exposure resulted in two- to eighty-fold increased concentrations of seven analytes with measurable baseline (vehicle control) levels, and increased concentration in eleven other analytes in which baseline levels were undetectable. ApoC-I co-exposure suppressed PIC-stimulated secretion of 12 of these analytes (Table 1). Interestingly, apoC-I co-exposure did not alter PIC-stimulated secretion of IFN-γ, IL-13, IP-10, MIG, MIP-1β, or RANTES. This is important mechanistically because it suggests that apoC-I-dependent reduction in PIC-stimulated cytokine release is cytokine-specific, and unlikely due simply to a reduction in the effective concentration of PIC via a direct interaction of PIC with apoC-I.

**Table 1:** Change in primary microglia response to 20 μg/ml PIC exposure in the presence or absence of 1 μM apoC-I

	**Concentration (pg/ml)**			**Change (%)**	
	**Vehicle**	**PIC**	**PIC/apoC-I**	**Vehicle vs. PIC**	**PIC vs. PIC/apoC-I**
G-CSF	ND	34.3	11.0	-	−68
IFN-γ	ND	12.8	9.3	-	−27
IL-1α	13.4	39.6	24.7	196	−38
IL-1β	ND	9.8	1.8	-	−82
IL-6	ND	795	28.9	-	−96
IL-13	ND	28.7	23.7	-	−17
IL-17	ND	13.2	8.6	-	−35
IP-10	ND	731.8	743.3	-	2
KC	ND	435.9	83.3	-	−81
LIX	83.6	193.7	99.9	132	−48
MCP-1	18.2	175.8	61	866	−65
M-CSF	4.3	9.4	6.8	119	−28
MIG	ND	604.6	599.1	-	−1
MIP-1α	41.2	2737.4	796.9	6544	−71
MIP-1β	ND	2128	1783.7	-	−16
MIP-2	38.2	3173.9	262.3	8209	−92
RANTES	ND	362	327.7	-	−9
TNF-α	7.4	409	124.4	5427	−70

WT murine primary microglia were exposed to 20 μg/ml PIC or 100 ng/ml LPS for 18 h in the presence or absence of 1 μM apoC-I, and supernatants were assayed with a 32-plex array for Eotaxin, granulocyte-colony stimulating factor (G-CSF), granulocyte macrophage-colony stimulating factor (GM-CSF), IFNγ, IL-1α, IL-1β, IL-2, IL-3, IL-4, IL-5, IL-6, IL-7, IL-9, IL-10, IL-12(p40), IL-12(p70), IL-13, IL-15, IL-17, interferon gamma-induced protein 10 (IP-10), keratinocyte-derived chemokine (KC), leukemia inhibitory factor (LIF), lipopolysaccharide-induced CXC chemokine (LIX), monocyte chemotactic protein-1 (MCP-1), macrophage-colony stimulating factor (M-CSF), monokine induced by gamma interferon (MIG), macrophage inflammatory protein (MIP)-1α, MIP-1β, MIP-2, regulated upon activation normal T-cell expressed and secreted (RANTES), TNF-α, and vascular endothelial growth factor (VEGF). PIC exposure resulted in two- to eighty-fold increased concentrations of seven analytes with measurable baseline (vehicle control) levels, and increased concentration in eleven other analytes in which baseline levels were undetectable. Of these, seven showed two- to eighty-fold induction over baseline (vehicle-control) levels and 11 others were induced for which there was no detectable baseline protein by PIC. ApoC-I co-exposure effectively reduced 12 of these PIC-stimulated cytokines. Data are expressed as cytokine amounts (pg/μg protein) from two independent experiments pooled from 3 replicate cultures and run in duplicate. ND: not detectable; PIC: polyinosinic-polycytidylic acid.

**Figure 3:**
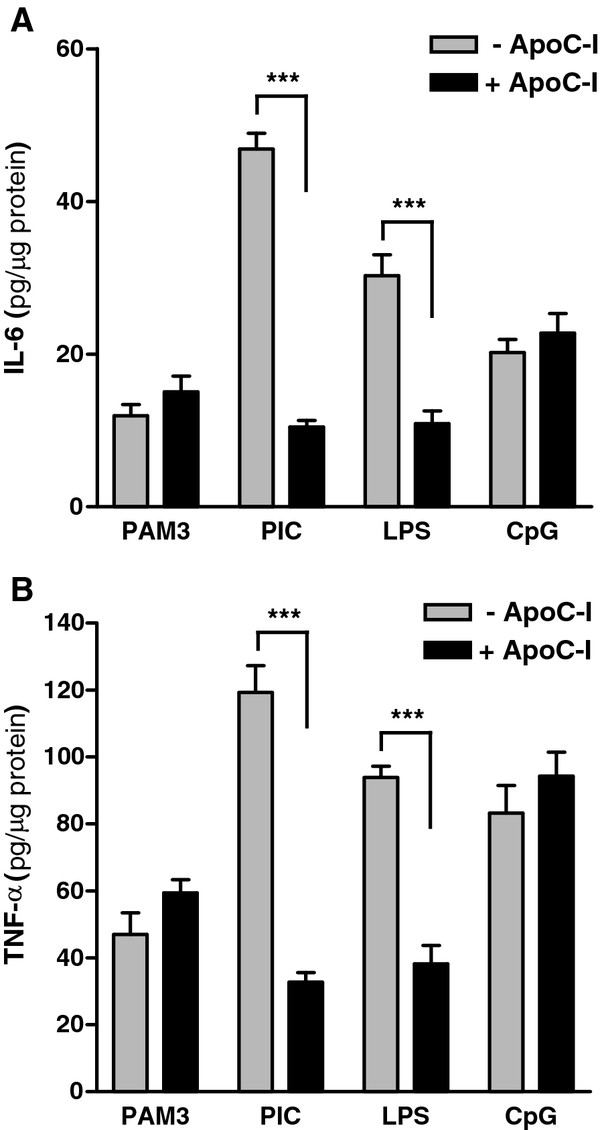
**ApoC-I suppresses toll-like receptor (TLR)-dependent activation of primary microglia.** Cultured WT primary murine microglia were treated with 1 μg/ml Pam3 (TLR2), 20 μg/ml PIC (TLR3), 100 ng/ml LPS (TLR4), or 1 μM CpG (TLR9) for 18 h in the presence or absence of 1 μM apoC-I and supernatant amounts of (**A**) IL-6 and (**B**) TNF-α (pg/μg protein) quantified by ELISA. ApoC-I significantly reduced PIC and LPS stimulated cytokine release from microglia, but had no effect on cells treated with Pam3 or CpG. Unstimulated vehicle controls were below the level of detection (data not shown). Data are expressed as the mean ± standard error of the mean (SEM) cytokine amount; n = 3 to 6. ***P < 0.001; analysis of variance (ANOVA) with Bonferroni’s multiple comparison test.

To validate the results of our screen, we chose to focus on two cytokines whose expression is stimulated by microglia in response to not only PIC but also Aβ_1-42_, IL-6 and TNF-α [40], and to broaden our activation paradigm. Using a panel of TLR activators (corresponding receptor), including PAM3 (TLR2), PIC (TLR3), LPS (TLR4), and CpG (TLR9), ELISA of conditioned medium confirmed PIC stimulation of IL-6 (Figure 3a) and TNF-α (Figure 3b) secretion from WT murine microglia after 18 h [39]. The amounts of both analytes in medium were below the level of assay detection in microglia treated with vehicle alone or 1 μM apoC-I (data not shown). Culture media from microglia that received PIC plus apoC-I or LPS plus apoC-I for 18 h had significantly reduced cytokine secretion compared with TLR agonist-only treatment (Figure 3a and b). Interestingly, no apoC-I immunosuppression was identified in cultures stimulated with either PAM3 or CpG (Figure 3a and b). These data suggest previously unidentified innate immunomodulatory activities of apoC-I that are specific to TLR3 and TLR4 activation.

**Figure 4:**
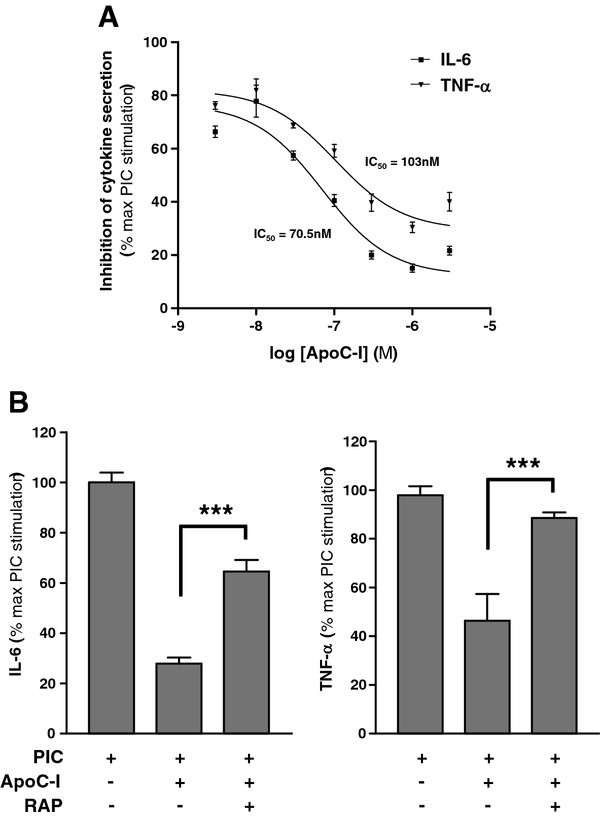
**ApoC-I suppression of toll-like receptor (TLR)-mediated microglial activation is dose and receptor-associated protein (RAP) dependent.** (**A**) Cultured WT primary mouse microglia were treated with 20 μg/ml PIC and various concentrations of ApoC-I as indicated for 18 h and supernatant amounts of IL-6 and TNF-α (pg/μg protein) quantified by ELISA. Data are expressed as mean ± standard error of the mean (SEM) percentage of the maximum PIC stimulation for each analyte; n = 3 to 5. (**B**) RAP significantly reversed apoC-I suppression of PIC-stimulated secretion of IL-6 and TNF-α by microglia. WT microglia were treated with 20 μg/ml PIC for 18 h in the presence of apoC-I (1 μM) and/or RAP (1 μM) and cytokine secretion measured by ELISA. Unstimulated vehicle controls were below the level of detection, and treatment with RAP alone did not stimulate cytokine release (data not shown). Data are expressed as mean ± SEM percentage of maximum PIC stimulation; n = 3 to 5. ***P < 0.001, analysis of variance (ANOVA) with Bonferroni’s multiple comparison test.

Because the most significant apoC-I-dependent reduction in TLR-mediated microglial activation was seen with PIC stimulation, we next investigated the concentration-dependence of this apoC-I effect. ApoC-I demonstrated concentration-dependent inhibition of PIC-stimulated secretion of IL-6 and TNF-α from primary WT murine microglia with an IC_50_ of 71 nM for IL-6 and 103 nM for TNF-α (Figure 4a). In fact, even < 10 nM apoC-I significantly suppressed PIC-induced secretion of IL-6 and TNF-α. Combined with the structural dissimilarities existing between TLR activators, the observation that apoC-I concentrations in molar excess to PIC only partially reversed microglial stimulation suggests that the mechanism of apoC-I suppression of cytokine secretion is independent of direct TLR agonist sequestration.

ApoC-I was originally found to directly antagonize receptor-mediated cellular uptake of particles containing apoE [36], an activity also ascribed to endogenous receptor associated protein (RAP) [41]. In order to further address potential mechanisms of apoC-I suppressor activity on stimulated microglia, we investigated the RAP-dependence of apoC-I immunosuppression. Co-exposure with RAP partially reversed the inhibitory effects of apoC-I on microglial secretion of both IL-6 (partially) and TNF-α (fully) (Figure 4b), while RAP alone had no effect on basal or PIC-induced cytokine secretion (data not shown). Taken together these data support an immunosuppressive action for apoC-I following TLR3 or TLR4 activation that is partially RAP-dependent, and unlikely to involve direct interaction between TLR activator and apoC-I.

**Figure 5:**
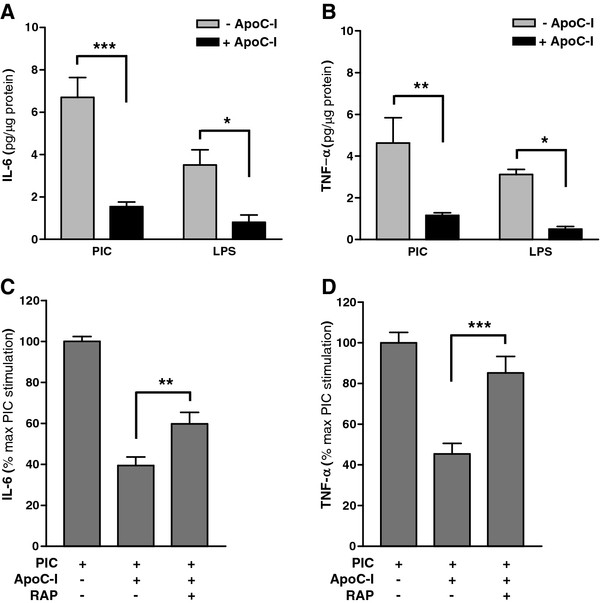
**ApoC-I suppresses TLR-dependent activation of primary astrocytes.** Cultured WT primary murine astrocytes were treated with 20 μg/ml PIC or 100 ng/ml LPS for 18 h in the presence or absence of 1 μM apoC-I and supernatant amounts of (**A**) IL-6 and (**B**) TNF-α (ng/μg protein) quantified by ELISA. ApoC-I significantly reduced PIC and LPS stimulated cytokine release from cultured astrocytes. Unstimulated vehicle controls were below the level of detection (data not shown). Data are expressed as mean ± standard error of the mean (SEM cytokine amount; n = 3 to 6. ***P < 0.001, analysis of variance (ANOVA) with Bonferroni’s multiple comparison test. (**C**,**D**) RAP significantly reversed apoC-I suppression of PIC-stimulated secretion of IL-6 and TNF-α by astrocytes. WT astrocytes were treated with 20 μg/ml PIC for 18 h in the presence of apoC-I (1 μM) and/or RAP (1 μM) and cytokine secretion measured by ELISA. Unstimulated vehicle controls were below the level of detection, and treatment with RAP alone did not stimulate cytokine release (data not shown). Data are expressed as mean ± SEM percentage of maximum PIC stimulation; *n* = 3. **P < 0.01; ***P < 0.001; ANOVA with Bonferroni’s multiple comparison test.

Like microglia, astrocytes also express functional TLRs, especially TLR3 and TLR4, and thus serve as sensitive innate immune effectors in brain [42] by secreting various pro-inflammatory cytokines in response to activation [43]. For this reason, we next investigated the possibility that apoC-I also may modulate TLR-dependent cytokine secretion from cultured primary murine astrocytes. As with activated microglia, apoC-I was able to partially suppress astrocyte response to PIC and LPS, significantly suppressing the release of IL-6 (Figure 5a) and TNF-α (Figure 5b). As expected, primary astrocyte cultures secreted lower levels of pro-inflammatory cytokines compared to microglia in response to the same TLR agonist concentrations; however, the apoC-I inhibitory effect on cytokine secretion was proportionately conserved between cell types. In addition, co-exposure to RAP reproduced observations made in microglia, partially to fully reversing the immunosuppressive effects of apoC-I on PIC-stimulated primary astrocyte cultures (Figure 5c and d). Likewise, basal and PIC-stimulated cytokine secretion by primary astrocytes was unaffected by RAP alone (data not shown).

### ApoC-I inhibits cytokine expression by human macrophages

**Figure 6:**
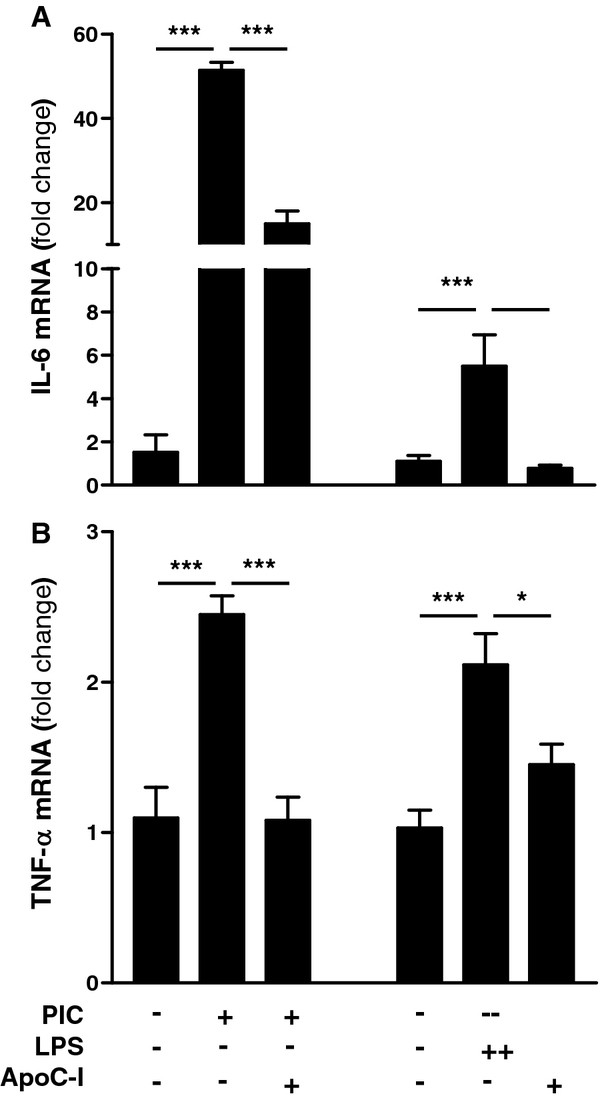
**Toll-like receptor (TLR)-dependent cytokine expression in human macrophages is suppressed by apoC-I.** Differentiated THP-1 cells were treated with 20 μg/ml PIC or 100 ng/ml LPS for 8 h in the presence or absence of 1 μM apoC-I and total RNA isolated from cells. qPCR analysis showed a significant stimulation of (**A**) IL-6 and (**B**) TNF-α mRNAs with both PIC and LPS treatment. Co-administration with apoC-I significantly reversed this effect. Data are expressed as mean ± standard error of the mean (SEM) fold induction; n = 3. *P < 0.05; **P < 0.01; ***P < 0.001, analysis of variance (ANOVA) with Bonferroni’s multiple comparison test.

To assess whether apoC-I modulates innate immune responses by human cells, we assessed the effect of apoC-I on IL-6 and TNF-α expression by the human acute monocytic leukemia cell line THP-1 [44]. For appropriate comparison to microglial experiments, THP-1 cells were differentiated into macrophage phenotype using the phorbol ester, PMA, prior to treatment [45]. Activation of differentiated THP-1 cells with either PIC or LPS resulted in a robust increase in expression of IL-6 and TNF-α mRNAs as expected (Figure 6a and b). Importantly, co-administration of cells with apoC-I significantly reduced TLR-dependent increases in both IL-6 and TNF-α expression, even to the point of completely blocking cytokine induction in some circumstances (Figure 6a and b). In addition to confirming previous results in human cells, these data also support a mechanism for apoC-I action at the transcriptional level and not strictly within the secretory machinery.

### ApoC-I reduces Aβ-induced cytokine secretion from microglia

**Figure 7:**
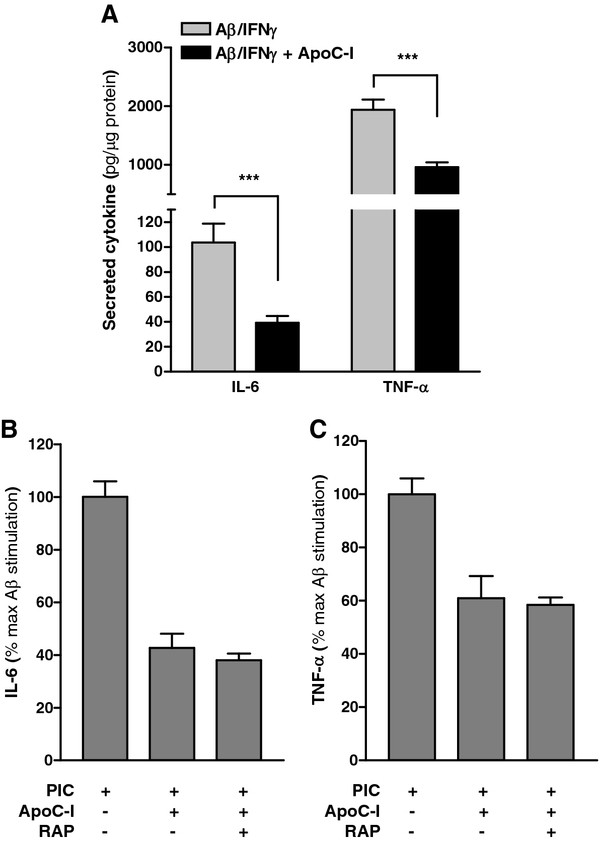
**ApoC-I suppresses Aβ-stimulated cytokine secretion from microglia.** Cultured WT primary murine microglia were treated with a combination of fibrillar Aβ_1-42_ (5 μM) and IFNγ (10U/ml) for 18 h and supernatant amounts of IL-6 and TNF-α (ng/μg protein) quantified by ELISA. Aβ/IFNγ induced robust secretion of IL-6 and TNF-α by cultured microglia into the extracellular media, an effect that was significantly reversed with co-administration of apoC-I (1 μM). Unstimulated vehicle controls were below the level of detection. Data are expressed as mean ± standard error of the mean (SEM) cytokine amount; n = 3. ***P < 0.001, Student’s *t*-test. (**B**,**C**) ApoC-I suppression of Aβ-stimulated secretion of IL-6 and TNF-α by microglia was unchanged by co-administration with RAP (1 μM). WT microglia were treated with Aβ_1-42_ and IFNγ for 18 h in the presence of apoC-I (1 μM) and/or RAP (1 μM) and cytokine secretion measured by ELISA. Data are expressed as mean ± SEM percentage of maximum PIC stimulation; n = 3.

Aβ deposition in brain represents the neuropathological hallmark of AD and is known to stimulate TNF-α and IL-6 secretion by cultured microglia [46]. Moreover, these cytokines can induce neuron stress in culture [47] and are identified in microglia localized with Aβ plaques in human brain [48]. Aβ is known to activate microglia through interaction with the CD14/TLR4 complex [49], and, in combination with interferon gamma (IFNγ), potently activates primary microglia to secrete neurotoxic cytokines [50], thus potentially exacerbating the inflammatory environment in diseased regions of AD brain. We exposed primary murine microglia to Aβ_1-42_ and IFN-γ according to commonly used protocols and measured subsequent cytokine secretion from culture medium (Figure 7a). As with PIC and LPS stimulation, apoC-I significantly reduced Aβ-induced microglia IL-6 and TNF-α secretion (Figure 7a). However, unlike microglia treated with PIC, co-exposure to RAP was ineffective in reversing the apoC-I-dependent suppression of IL-6 and TNF-α secretion from Aβ-stimulated microglia (Figure 7b and c). Taken together, these results indicate that apoC-I may partially suppress Aβ-dependent neuroinflammation through a RAP-independent mechanism, confirming previous results indicating that the mechanisms of Aβ-induced microglial activation overlap only partially with canonical TLR activators [17].

## Discussion

*APOE* represents the most significant genetic risk factor for development of sporadic AD, and this very strong genetic association highlights the molecular relevance of *APOE* in AD etiology and/or pathogenesis. However, molecular relevance does not define mechanism of action. Since *APOE* is known to regulate expression of other neighboring genes [35,51], including *APOC1*, we hypothesized that immune regulation by apoC-I might be one mechanism underlying the association between *APOE* and immune response in mice and humans.

We show for the first time that CSF levels of apoC-I from normal, aged human subjects are dependent partially on *APOE* genotype, where the presence of an *APOE* ϵ4 allele is associated with significantly reduced apoC-I compared with individuals homozygous for *APOE* ϵ3. We experimentally tested this genetic association using two approaches. First, we demonstrated in TR APOE mice that apoC-I mRNA from liver and brain, along with apoC-I serum protein concentration, are lower in TR APOE4 than in TR APOE3 mice. In addition, we showed that primary cerebral cortical astrocytes cultured from TR APOE4 mice had reduced apoC-I mRNA compared to TR APOE3 cultures, also validating CNS *de novo* synthesis of apoC-I by astrocytes [26]. Our data also support a direct influence of the *APOE* ϵ4 allele or isoform on apoC-I expression independent of *APOC1* polymorphism since the targeted replacement mice differ only in *APOE* genotype and not *APOC1* haplotype (that is, H2 polymorphism). While our data cannot resolve the apparently complex interaction between *APOE* and *APOC1*, we note that the single nucleotide within exon 4 that distinguishes TR APOE4 from TR APOE3 has intrinsic enhancer activity and may partially account for *APOE*-dependent expression differences seen among genes in the *APOE/C-I/C-IV/C-II* cluster [52]. In addition, while little is known about the complex intrinsic regulation of the *APOE/C-I/C-IV/C-II* cluster, genetic deletion of *APOE* and *APOC1* individually or collectively leads to disrupted gene expression of cluster members [35].

Regardless of the exact mechanism by which *APOE* influences expression of *APOC-I* in mice and humans, we have experimentally demonstrated a novel innate immunosuppressive action of apoC-I. Using a 32-plex screen of inflammatory proteins secreted from PIC-stimulated microglia, we identified multiple cytokines, including TNF-α and IL-6, which had their expression and/or secretion suppressed by apoC-I in a concentration-dependent manner. Not all cytokines secreted in excess following microglia activation were suppressed by apoC-I, arguing strongly against direct interaction of apoC-I with TLR activator. Microglia can be stimulated to adopt a pro-inflammatory, M1 phenotype, resulting in secretion of cytotoxic reactive oxygen species and nitric oxide, as well as various cytotoxic cytokines, including TNF-α and IL-6 (reviewed in [53]), not only by specific TLR activators but also by Aβ [54]. Aβ-stimulated proinflammatory cytokine secretion from wild-type microglia was effectively suppressed by apoC-I co-administration in our primary culture system. We have found that the coordinated response of microglia to innate immune activation is modulated by apoC-I, with an inverse relationship between the concentration of apoC-I and potentially neurotoxic cytokines. It is important to note that although we used appropriately lipidated peripheral human apoC-I for our experiments, the immunosuppressive activities of apoC-I should be confirmed *in vivo* to account for the possibility of differentially lipidated lipoprotein pools synthesized in the CNS. Moreover, the concentration of ApoC-I measured in human lumbar fluid (Figure 1) ranged from about 50 nM to 200 nM; other proteins and neurotransmitters produced in brain have an approximately 10- to 100-fold greater concentration in extracellular fluid of brain compared with CSF in the lumbar sac. Therefore, although not directly determined using techniques such as microdialysis, the 1 μM ApoC-I used in our cell culture experiments is expected to be close to the physiologic ApoC-I concentration in extracellular fluid of brain. We speculate that individuals with at least one *APOE* ϵ4 allele may experience increased neuronal stress resulting from enhanced chronic activation of innate immune response because of reduced apoC-I expression.

Rensen and colleagues have published numerous *in vitro* and *in vivo* studies showing that apoC-I directly interacts with LPS to enhance pro-inflammatory innate immune actions of peripheral effectors directed toward elimination of bacterial infection [55-57]. Furthermore, these investigators have associated these findings to clinical outcomes by showing that plasma apoC-I levels positively correlate with improved outcome in elderly patients at risk for infection [57-59]. Our findings do not support a significant contribution of direct interaction of apoC-I with immune activator in our experiments. Indeed, our results showing that (1) apoC-I suppressed glial activation in response to three structurally distinct stimulants (LPS, PIC, and Aβ), (2) the saturable kinetics of interaction, and (3) selectivity for some, but not all, induced cytokines/chemokines strongly argue against direct interaction of apoC-I with immune activator. Peripheral actions of apoC-I are mediated in part through direct interaction with LDL receptor-related protein (LRP), including inhibition of apoE-dependent cellular uptake of lipoprotein [36]. RAP is also known to inhibit LRP-dependent processes [41], and so the partial RAP-dependence of apoC-I immunosuppressive activity on PIC-stimulated cells, but not microglia treated with Aβ, suggests a more complex yet unidentified mechanism of action, one where apoC-I has suppressive effects independent of direct interaction with the inflammatory stimulants. Despite these reasons, even if apoC-I immunosuppressive activity is partially due to direct interaction with diverse innate immune activators, inhibition of neurotoxicity by binding to a biologically relevant peptide such as Aβ would have important clinical implications.

There is little doubt that apoC-I plays a critical role in CNS homeostasis, since both the overexpression or absence of apoC-I in mice impairs memory [27,60]. We interpret these data to demonstrate the important role played by balanced apoC-I expression in CNS function. Consistent with our results, IL-6 and TNF-α expression were increased in the brains of apoC-I knockout mice [60]. These reports, in combination with our data, suggest that aberrant modulation of apoC-I in the diseased brain may provide a novel therapeutic target not only for AD but also for other neurodegenerative diseases in which the innate immune response contributes to neuronal stress and degeneration. Future experiments are needed to determine whether peripheral modulation of apoC-I can effectively mitigate clinical and neuropathologic outcomes of AD.

## Conclusions

We have identified a novel immunosuppressive activity of apoC-I and have confirmed reduced expression of apoC-I in the context of *APOE* ϵ4 genotype in mice and humans. ApoC-I immunosuppression in the presence of diverse innate immune activators, selective modulation of cytokine and chemokine secretion, reaction kinetics, and the selective partial RAP-dependence suggest a mechanism at least partially independent of apoC-I interactions with innate immune effectors. ApoC-I immunosuppression in the context of Aβ innate immune activation is potentially clinically relevant and perhaps provides a novel therapeutic target for AD and other neurodegenerative diseases in which innate immune activation contributes to neuronal damage. Overall, these data raise the possibility that altered levels of apoC-I might be one mechanism by which inheritance of different *APOE* alleles modulate CNS innate immune response.

## Abbreviations

Aβ: amyloid β; AD: Alzheimer’s disease; apo: apolipoprotein; *APOE*: human gene for apoE; CNS: central nervous system; CSF: cerebrospinal fluid; DMEM: Dulbecco’s modified Eagle’s medium; ELISA: enzyme-linked immunosorbent assay; FBS: fetal bovine serum; GAPDH: glyceraldehyde 3-phosphate dehydrogenase; G-CSF: granulocyte-colony stimulating factor; GM-CSF: granulocyte macrophage-colony stimulating factor; HDL: high density lipoprotein; IFN: interferon; IL: interleukin; IP-10: interferon gamma-induced protein 10; lrpα: receptor-related protein; KC: keratinocyte-derived chemokine; LDL: low density lipoprotein; LIF: leukemia inhibitory factor; LIX: lipopolysaccharide-induced CXC chemokine; LPS: lipopolysaccharide; MCP-1: monocyte chemotactic protein-1; M-CSF: macrophage-colony stimulating factor; MIG: monokine induced by gamma interferon; MIP: macrophage inflammatory protein; Pam3: Pam_3_CSK_4_; PIC: polyinosinic-polycytidylic acid; PMA: phorbol 12-myristate; 13-acetate; RANTES: regulated upon activation, normal T-cell expressed, and secreted; RAP: receptor associated protein; THP-1: human acute monocytic leukemia cell line; TLR: toll-like receptor; TR: targeted replacement; TNF: Tumor necrosis factor; VEGF: vascular endothelial growth factor; VLDL: very low density lipoprotein; WT: Wild type.

## Competing interests

The authors declare that they have no competing interests.

## Authors’ contributions

EC, XL, and TY carried out assays of human CSF, experiments with mouse cells. EC participated in the design of the study and drafted the manuscript. SC performed clinical research to obtain human CSF. SC and TM performed statistical analysis. KM, TM, and CK conceived the study, participated in its design and coordination, and helped to draft the manuscript. All authors read and approved the final manuscript.
